# Identification of Circulating MicroRNAs as Potential Biomarkers for Detecting Acute Myeloid Leukemia

**DOI:** 10.1371/journal.pone.0056718

**Published:** 2013-02-20

**Authors:** Feng Zhi, Xiangshan Cao, Xiaobao Xie, Biao Wang, Weimin Dong, Weiying Gu, Yun Ling, Rong Wang, Yilin Yang, Yan Liu

**Affiliations:** 1 Modern Medical Research Center, Third Affiliated Hospital of Soochow University, Changzhou, Jiangsu, China; 2 Department of Hematology, Third Affiliated Hospital of Soochow University, Changzhou, Jiangsu, China; 3 Department of Neurosurgery, Third Affiliated Hospital of Soochow University, Changzhou, Jiangsu, China; University of Nevada School of Medicine, United States of America

## Abstract

Acute myeloid leukemia (AML) is the most common acute leukemia in adults. The disease is characterized by various cytogenetic and molecular abnormalities with distinct prognoses and gene expression profiles. Emerging evidence has suggested that circulating microRNAs (miRNAs) could serve as noninvasive biomarkers for cancer detection; however, little is known about circulating miRNA profiles in AML patients. In this study, a genome-wide serum miRNA expression analysis was performed using Solexa sequencing for initial screen, followed by validation with real-time PCR assays. The analysis was conducted on training and verification sets of serum samples from 140 newly diagnosed AML patients and 135 normal adult donors. After a two-phase selection and validation process, 6 miRNAs, miR-10a-5p, miR-93-5p, miR-129-5p, miR-155-5p, miR-181b-5p and miR-320d, were found to have significantly different expression levels in AML compared with control serum samples. Furthermore, unsupervised clustering analysis revealed the remarkable ability of the 6-miRNA profile to differentiate between AML patients and normal controls. The areas under the ROC curve for the selected miRNAs ranged from 0.8129 to 0.9531. More importantly, miR-181b-5p levels in serum were significantly associated with overall survival. These data demonstrated that the expression patterns of circulating miRNAs were systematically altered in AML and miR-181b-5p may serve as a predictor for overall survival in AML patients.

## Introduction

Acute myeloid leukemia (AML), the most frequent hematological malignancy in adults, is characterized by an accumulation and differentiation arrest of myeloid blasts in the bone marrow and blood that requires immediate treatment to prevent interference with the production of healthy white blood cells in the bone marrow. The French-American-British (FAB) classification system divides AML into 8 subtypes, M0 through M7, based on the type of cell from which the leukemia developed and the cell’s degree of maturity [1]. Indeed, the treatment choice and prognosis for newly diagnosed AML patients are based mainly on cytogenetic information, which classifies AML into three risk-based categories: favorable, intermediate, and poor. The favorable prognosis, with a 5-year overall survival (OS) rate of 55%, is associated with AML patients carrying t(16;16), t(15;17) or t(8;21). The intermediate subgroup has a 5-year OS rate ranging between 24 and 42% and includes patients with normal cytogenetics, trisomy 8 or t(9;11). Patients with -5, -5q, -7, -7q, 11q23, t(3;3), t(6;9), t(9;22) or complex cytogenetics are classified as having a poor prognosis, and the 5-year OS rate is only approximately 11% [2]. Despite intensive research in recent decades, the cause of AML is not yet fully understood, and better prognostic indicators and more effective targeted therapies remain elusive.

MicroRNAs (miRNAs) are small non-coding RNAs of 19–24 nucleotides in length that regulate gene expression by base pairing with the 3′-untranslated region of a target gene’s mRNA, leading to degradation and/or translational repression of that gene [3]. miRNAs have been implicated in many biological events, and their deregulation is associated with leukemogenesis. Many miRNA expression studies have been performed to identify miRNAs that are differentially expressed between normal and leukemic samples [4], [5], [6]. Recently, miRNAs have been demonstrated to be present in serum or plasma in a stable and reproducible fashion, and the unique expression patterns of serum or plasma miRNAs can be used as fingerprints for various diseases [7], [8]. However, the global serum miRNA pattern in AML patients has not yet been reported. In this study, we employed high-throughput Illumina Solexa sequencing scanning, followed by a stem-loop quantitative reverse-transcription PCR (qRT-PCR) assay, to systematically and extensively investigate the serum miRNA expression profiles in AML.

## Results

### Solexa Sequencing of Serum miRNAs in AML

**Figure 1:**
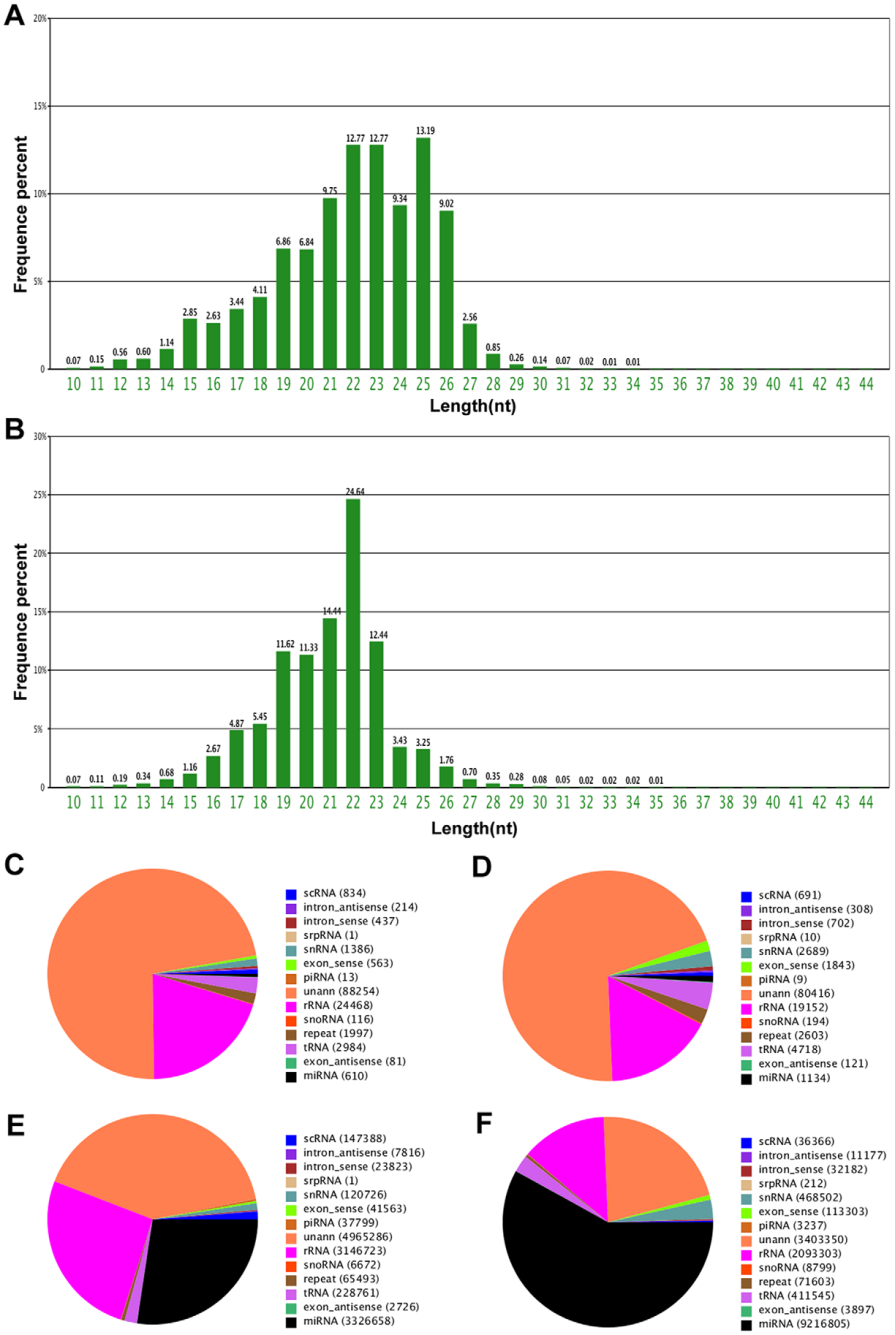
The circulating miRNAs signatures identified by solexa sequencing. The length distribution and frequency percentages of the sequences identified in normal subjects (A) and AML patients (B); RNA species in normal subjects (C) and AML patients (D); and RNA read counts in normal subjects (E) and AML patients (F).

To select candidate serum miRNAs for AML detection, we performed an initial genome-wide miRNA screening of two pools of serum samples derived from 20 AML patients and 20 matched normal controls by Solexa sequencing (1.0 mL for each sample, 20 mL total for each group). For the sequences in the two libraries, the majority of the small RNAs were 19–24 nt long, which was typical of Dicer-processed products. In the control library, 22 nt and 23 nt RNAs were the dominant small RNAs, each accounting for 12.77% of the total sequences (Fig. 1A), while 22 nt RNAs accounted for 24.64% of the total sequences in the AML library (Fig. 1B). Although miRNAs represented only a tiny fraction of the total small RNAs in both the control and AML serum samples, the expression levels of the individual miRNAs were relatively high (Fig. 1C–1F). Furthermore, the number of unique miRNA sequences and the amount of miRNA species were higher in the AML patients compared with the normal controls (1134 vs. 610 and 9216805 vs. 3326658, respectively). The miRNA levels were considered to be differential only if Solexa sequencing detected at least 50 copies in the control group and identified at least a 2-fold change between the two pooled samples. Among the miRNAs detected, 270 miRNAs were found to be at differential levels in AML sera compared with normal sera, of which 96 were downregulated and 174 were upregulated (Table S1).

### qRT-PCR Analysis of Serum miRNAs in AML

We confirmed the concentrations of the 25 candidate miRNAs, which were selected following Solexa sequencing with a hydrolysis probe-based qRT-PCR assay of the samples from 140 patients with a clinical and pathologic diagnosis of AML and from 135 healthy control individuals. The criteria for selecting the miRNA for qRT-PCR analysis were the following: 1) Solexa reads >50; 2) fold change >2; 3) miRNA number <500; 4) miRNA star is omitted; and 5) Cq value <35. Consequently, 25 miRNAs that met the inclusion criteria were chosen for further analysis. We found no significant differences between the cancer patients and control individuals in age and gender distribution.

Within the training set, the miRNAs of individual serum samples from 45 AML patients and 40 healthy controls were measured, and only miRNAs with a mean change ≥2.0-fold and a *p*-value <0.05 were selected for further analysis. Moreover, miRNAs with a Cq value >35 and a detection rate <75% in either the AML group or the control group were excluded from further analysis. We used these criteria to generate a list of 11 miRNAs (miR-10a-5p, miR-22-3p, miR-93-5p, miR-129-3p, miR-129-5p, miR-139-5p, miR-155-5p, miR-181b-5p, miR-320d, miR-375 and miR-485-5p) that showed a difference in miRNA patterns between AML patients and controls (Table 1).

**Table 1:** Serum microRNAs differentially expressed in AML samples compared to normal controls.

miRNA	training set		validation set		training+validation	
	Fold change (mean±SE;AML/normal)	p-value	Fold change (mean±SE;AML/normal)	p-value	Fold change (mean±SE;AML/normal)	p-value
miR-10a-5p	3.81±0.29	4.98×10^−13^	2.23±0.27	2.34×10^−5^	2.55±0.22	8.16×10^−10^
miR-93-5p	3.40±0.14	1.12×10^−18^	4.48±0.34	4.78×10^−20^	4.11±0.24	5.76×10^−28^
miR-129-5p	3.28±0.29	5.84×10^−10^	3.46±0.20	5.90×10^−22^	3.41±0.16	2.24×10^−29^
miR-155-5p	6.11±0.73	2.69×10^−9^	4.42±0.20	1.05×10^−37^	4.79±0.23	6.73×10^−39^
miR-181b-5p	2.29±0.29	7.00×10^−5^	2.27±0.21	8.17×10^−7^	2.31±0.17	2.42×10^−10^
miR-320d	7.60±0.88	3.37×10^−10^	5.02±0.29	8.17×10^−7^	5.53±0.29	1.22×10^−33^
miR-22-3p	3.24±0.27	1.56×10^−11^	1.54±0.32	3.52×10^−9^		
miR-129-3p	2.18±0.13	2.15×10^−12^	1.38±0.37	2.17×10^−25^		
miR-139-5p	4.85±0.47	5.77×10^−11^	1.81±0.15	7.55×10^−15^		
miR-375	3.08±0.29	1.23×10^−8^	1.42±0.19	4.32×10^−8^		
miR-485-5p	4.26±0.53	8.06×10^−8^	1.63±0.28	6.33×10^−4^		

**Figure 2:**
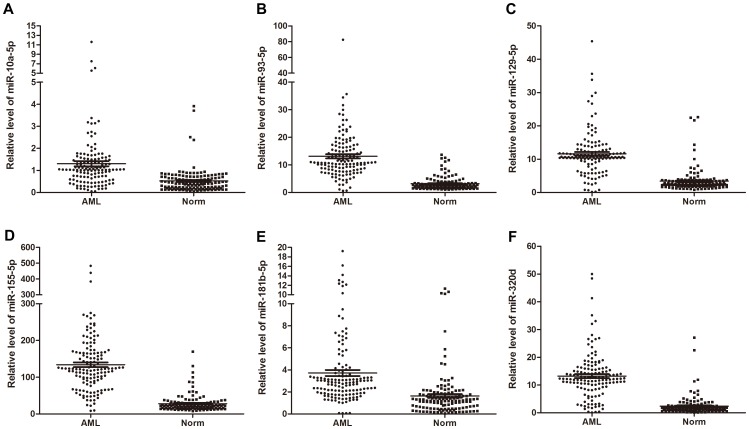
The relative levels of 6 serum miRNAs in 140 AML patients and 135 healthy control individuals (in both the training and validation sets) determined using a qRT-PCR assay. Each point represents the mean results for triplicate samples.

To verify the accuracy and specificity of these miRNAs and to refine the number of miRNAs to be used as the AML signature, we further examined these 11 miRNAs in the validation set, which consisted of 95 AML patients and 95 normal controls, by qRT-PCR. The miRNAs were considered to be significantly differentially expressed only when they exhibited a mean change >2- or <0.5-fold relative to the controls, a *p*-value <0.05, and a parallel trend in variation between the AML and control groups in both the training set and the validation set. Our analysis ultimately generated a list of 6 miRNAs, consisting of miR-10a-5p, miR-93-5p, miR-129-5p, miR-155-5p, miR-181b-5p, and miR-320d, that were differentially expressed in the AML patients compared with normal controls (Table 1). The changes in concentration ranged from 2.31- to 5.53-fold (Table 1). Fig. 2 shows the differences in concentration for the 6 miRNAs in the 140 AML patients and 135 control individuals enrolled in the training and validation sets.

### Unsupervised Clustering Analysis and ROC Curve Analysis

We analyzed the differential expression of miRNAs between the AML and normal control serum samples by performing unsupervised clustering that was blinded to the clinical annotations. The dendrogram generated by the cluster analysis showed a clear separation of the AML samples from the control samples based on the serum 6-miRNA profile (miR-10a-5p, miR-93-5p, miR-129-5p, miR-155-5p, miR-181b-5p, and miR-320d). In the training set, only 7 of the 40 (17.5%) control samples and 1 of the 45 (2.2%) AML samples were misclassified (Fig. S1A). In the validation set, 95 AML samples and 95 normal controls were also clearly separated into two main classes, with only 4 (4.2%) AML samples and 11 (11.6%) control samples misclassified (Fig. S1B). Finally, a similar result was obtained when we mixed samples from the training and validation sets, as only 8 (4.2%) AML samples and 10 (11.6%) control samples were classified incorrectly out of 140 AML and 135 control samples (Fig. S1C).

**Figure 3:**
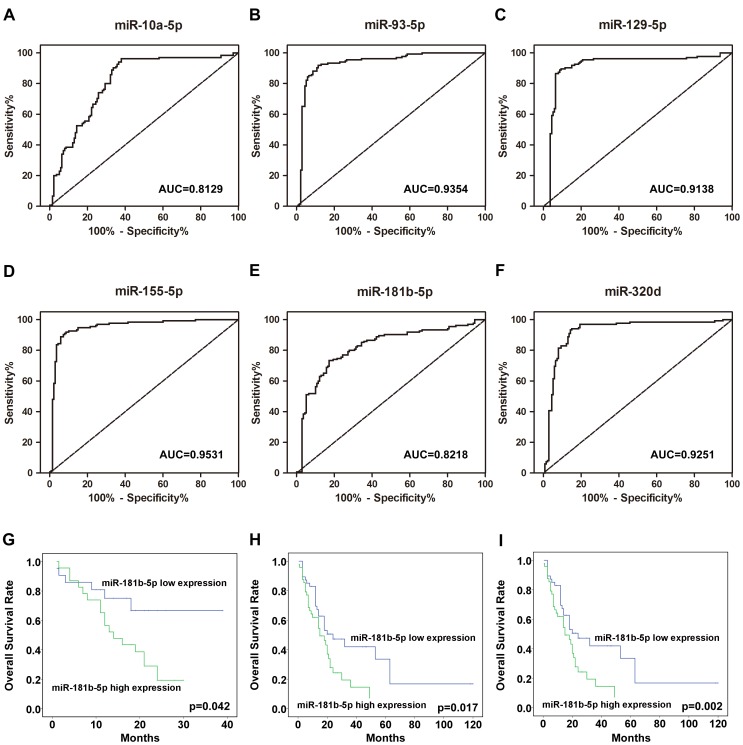
The ROC curves reflecting the ability of the serum levels of the 6 individual miRNAs (A-F), miR-10a-5p, miR-93-5p, miR-129-5p, miR-155-5p, miR-181b-5p, and miR-320d, to differentiate the AML cases (n = 140) from the controls (n = 135); Kaplan-Meier estimates of the overall survival of AML patients in the miR-181b-5p high-expression group compared with the miR-181b-5p low-expression group in the training set (G), in the validation set (H), and in both the training and validation sets (I).

The ROC curves constructed to compare the relative concentrations of the 6 miRNAs for the AML patients and healthy controls yielded the following AUCs: miR-10a-5p, 0.8129 (95% confidence interval (CI) = 0.7610–0.8649); miR-93-5p, 0.9354 (95% CI = 0.9028–0.9681); miR-129-5p, 0.9138 (95% CI = 0.8724–0.9552); miR-155-5p, 0.9531 (95% CI = 0.9259–0.9803); miR-181b-5p, 0.8218 (95% CI = 0.7703–0.8733); and miR-320d, 0.9251 (95% CI = 0.8892–0.9610) (Figs. 3A–3F). These results suggest the potential of these miRNAs for discriminating patients with AML from healthy subjects.

### Correlation between Serum miRNA Expression Profiles and Overall Survival Rate of Patients with AML

We next investigated the correlation between the serum miRNA expression profiles and the overall survival outcome using prospective follow-up data collected from the 140 AML patients. Due to the observation that 6 miRNAs were differentially expressed between AML and normal controls, these miRNAs were evaluated as candidates for prognosis determination. The expression levels of these 6 miRNAs in AML were first stratified by the median value, and the overall survival outcome of patients with high miRNA expression levels (greater than the median) was then compared with those of patients with low miRNA expression levels (less than the median), as determined by Kaplan-Meier survival analysis. We first analyzed the follow-up data collected from the 45 patients in the training set. We observed a worse overall survival rate that was marginally significant in AML patients showing high levels of miR-181b-5p (p = 0.042; log-rank test) (Fig. 3G). To validate the findings from the training set, miR-181b-5p levels were assessed using the follow-up data collected from 95 patients in the validation set, and we observed a consistent result (Fig. 3H). More impressively, when we analyzed the follow-up data from the training set and the validation set together, miR-181b-5p showed a significant correlation with the overall survival outcome (Fig. 3I). Taken together, the results suggest that the expression of miR-181b-5p may have prognostic value for AML patients.

## Discussion

Many research groups have reported on the close relationship between aberrant miRNA expression and the pathogenesis, diagnosis and prognosis of AML [9], [10], [11]. However, the role of serum miRNA in AML has not been thoroughly studied. Serum samples are easily acquired in a relatively noninvasive manner, and isolated miRNAs are readily detected by qRT-PCR, a widely used clinical and laboratory technique. Therefore, the determination of the serum miRNA profiles in AML patients is quite meaningful. In the present study, we analyzed the circulating miRNAs in AML patients of different subtypes ranging from M1 to M5. By performing an initial genome-wide Solexa survey followed by two sets of individual qRT-PCR evaluations, we identified 6 serum miRNAs, miR-10a-5p, miR-93-5p, miR-129-5p, miR-155-5p, miR-181b-5p and miR-320d, whose concentrations were significantly upregulated in the serum of AML patients compared with normal controls. Unsupervised hierarchical clustering, together with ROC curves, revealed that the 6-miRNA panel has a promising ability to distinguish AML individuals from normal healthy controls. Furthermore, miR-181b-5p was identified as a potential prognostic factor that was significantly associated with prolonged overall survival.

The discovery that serum miRNAs can serve as potential biomarkers overcomes the problem of collecting tissue samples through invasive procedures such as biopsy or surgery [7], [8]. A serum miRNA-based biomarker would allow the comprehensive analysis of diseases in a less invasive manner. The Solexa sequencing assay is an effective high-throughput tool for initial serum miRNA screening with high accuracy and sensitivity. However, the Solexa results should be validated by qRT-PCR using a large number of individual serum samples, as the Solexa results are obtained from pooled serum samples and might therefore include inaccurate information due to individual variation. In the process of searching circulating miRNA-based cancer biomarkers, researchers have developed a working model to identify and refine differentially expressed miRNAs in cancer serum/plasma samples compared with control samples. The analysis was separated into three steps: (i) initial screening by high-throughput techniques such as Solexa sequencing, microarray, or miRNA cloning using pooled or several serum samples; (ii) qRT-PCR validation in a small number of individual serum samples arranged in a training set; and (iii) qRT-PCR validation in a large number of individual serum samples arranged in a testing set [12]. Cross-validation, or rotation estimation [13], is a technique used assess whether the results of a statistical analysis will generalize to an independent data set and to determine how accurately a predictive model will perform in practice. A single round of cross-validation involves partitioning the data sample into complementary training and validation subsets, performing the analysis on the training subset, and validating the analysis on the other subset. To reduce variability, multiple rounds of cross-validation are performed using different partitions, and the validation results are averaged for all the rounds. This model has proven to be greatly successful in identifying serum miRNA-based biomarkers for cancer classification [14], [15]. In our study, 174 miRNAs were upregulated, as determined from an initial screening by Solexa sequencing, but only 6 miRNAs exhibited statistically significant positive differences in AML after multiple rounds of qRT-PCR validation using a large number of individual samples.

Many published reports have demonstrated specific miRNA expression profiles associated with different types of or cytogenetically/molecularly distinct AMLs [16]. Here, we also tested whether our serum miRNA-based biomarkers were applicable to the precise clinical classification of cytogenetically/molecularly distinct AMLs. However, no obvious differences were observed when the AML cases were stratified by AML subtypes. For example, when AML samples were stratified by FAB subtype (M1 to M5), no obvious differences were observed, as miR-93-5p, miR-129-5p, miR-155-5p and miR-320d were all upregulated in each subtype of AML (Table S2). This result may have been obtained because our initial screening was performed using AML serum samples without subtype separation. Thus, the 6-serum miRNA profile was unable to absolutely distinguish AML subtypes from each other. Future studies may require the direct comparison of sera from different histological subtypes of AML at the miRNA screening phase.

miRNAs are known to play crucial roles as oncogenes or tumor suppressors, and their deregulation is involved in multiple processes including cell proliferation, apoptosis, cell-cycle regulation and invasion in various diseases. Among the 6 serum miRNAs identified in this study, some have already been reported to play important roles in AML. For example, miR-10a-5p is aberrantly overexpressed in Nucleophosmin1 (NPM1)-mutated AML and may exert its biological properties in AML by interfering with the p53 machinery, which is partly regulated by MDM4 [17], [18]. The upregulation of miR-155-5p is observed in AML patients with FLT3 internal tandem duplications (ITD), which is associated with an adverse clinical outcome [19], [20], [21], [22]. Additionally, overexpression of miR-155-5p causes a myeloproliferative disorder in hematopoietic stem cells [23]. However, there are no reports about the role of miR-93-5p, miR-129-5p and miR-320d in AML. In laryngeal squamous cell carcinoma and in cisplatin-resistant ovarian cancer cells, miR-93-5p is significantly upregulated and may promote tumor growth and angiogenesis by targeting integrin-β8 [24], [25], [26]. In colon cancer stem-like cells, miR-320d is overexpressed and might play important roles in the maintenance of colon cancer stem cell stemness [27]. The decreased expression of miR-129-5p is frequently observed in multiple tumor cell lines and primary tumors, and it inhibits cell proliferation and induces cell death by targeting different genes, such as GALNT1, SOX4, Cdk6 and VCP/97 (valosin containing protein) [28], [29], [30]. Recently, miR-129-5p was reported to be underexpressed in human hematopoietic stem cells, and EIF2C3 and CAMTA1 were identified as its targets, indicating that miR-129-5p may also play an important role in the genesis of AML [31]. miR-181b-5p typically functions as a tumor suppressor in different tumors, and the restoration of miR-181b-5p expression could significantly promote apoptosis, inhibit proliferation of leukemic cells and delay leukemogenesis [10]. Until recently, only a small subset of genes has been identified as miR-181b-5p target genes: p27, CREB1 and TIMP3 are involved in cell proliferation [32], [33], [34], [35]; BCL-2, MCL-1 and XIAP are associated with apoptosis and chemoresistance [36], [37]; Nrp1 is involved in cell migration [38], and importin-α3 is involved in vascular inflammation [39]. Most of the miR-181b-5p target genes are associated with cell proliferation, apoptosis and chemoresistance, indicating the important role that miR-181b-5p may play in the genesis of AML.

miR-129-5p and miR-181b-5p are frequently downregulated in cell lines or primary tumors, but in the present study, they were found to be upregulated in AML serum. The discrepancy between the expression patterns of circulating miRNAs and tissue/cellular miRNAs previously identified in the same type of tumor suggests that the origin of circulating miRNAs is complicated. Circulating miRNAs have been suggested to result from passive leakeage from broken cells or actively secreted from living cells via exosomes and shedding vesicles [12]. Generally, there is a close relationship between tissue miRNAs and circulating miRNAs. For example, the expression of miR-25 and miR-223 has been found to be increased in both the serum of lung cancer patients [7] and their lung tumor tissues [40]. The level of miR-155 expression has also been found to be elevated in the tumor tissues/cells [41] and plasma of lymphoma patients [42]. However, many miRNAs show the opposite trend, with disparate expression levels in the plasma/serum and tumor tissues of patients with various types of cancer. For example, miR-145 was found to be downregulated in NSCLC tissues [43], though it was upregulated in NSCLC serum [44]. Thus, the expression patterns between tissue and circulating miRNAs may differ, and the underlying mechanism for this disparity is still controversial. Recently, Ohshima et al. revealed that the let-7 miRNA family was enriched in the extracellular exosomes of a metastatic gastric cancer cell line AZ-P7a, while AZ-521 cells with low metastatic potential showed no such propensity [45]. Because let-7 miRNAs generally function as tumor suppressor genes to target oncogenes such as RAS and high mobility group A2 (HMGA2), they proposed that AZ-P7a cells selectively secrete let-7 miRNAs into the extracellular environment via exosomes to reduce the anti-tumorigenic effect within the cells and maintain their oncogenesis [45]. Because miR-129-5p/miR-181b-5p generally functions as a tumor suppressor (although the current study did not provide direct evidence demonstrating the origin of miR-129-5p/miR-181b-5p), we herein propose that tumor cells may secrete miR-129-5p/miR-181b-5p into the extracellular environment via exosomes to reduce the anti-tumorigenic effect within the cells, leading the tumor cells to maintain their oncogenic and metastatic propensities. Thus, although the expression patterns of miR-181b-5p did not overlap between serum and tissue, the serum miR-181b-5p expression level may also reflect various aspects of the human physiological status and serve as fingerprints for AML diagnosis.

The prognostic factors for acute leukemia have experienced major changes over the past decade and are likely to be further refined in the coming years. While age is the single most important prognostic factor in both AML and acute lymphoblastic leukemia (ALL), recurring cytogenetic abnormalities and molecular markers have become crucial for predicting remission rate, relapse, and OS in AML. Mutations in FLT3, NPM1, CEBPA, KIT (CD117), IDH1, and IDH2 and the overexpression of BAALC, MN1, and ERG1 have been identified as having prognostic significance in AML [46]. The emergence of miRNA research opens a new venue for early and accurate determination of AML pathogenesis, diagnosis and prognosis. For example, high miR-29b expression was correlated with a favorable overall survival and effective clinical response to decitabine in AML patients [47], [48]. The miR-181 family expression signature was associated with *CEBPA* mutational status, and the miR-181a-5p and miR-181b-5p expression levels were inversely associated with event-free survival in cytogenetically normal AML patients with high molecular risk [4], [49]. The expression levels of miR-181a were strongly associated with clinical outcomes in cytogenetically normal AML patients independent of other established clinical and molecular predictors [50]. In this study, miR-181b-5p was significantly upregulated in AML serum samples, and higher expression levels of miR-181b-5p were correlated with a poorer overall survival outcome. Because the levels of miR-181b-5p in serum and tissue are differentially correlated with patient survival, further studies are required to determine the origin of circulating miR-181b-5p, as well as its biological relevance.

In this study, a serum 6-miRNA-based expression profile that was able to accurately discern AML individuals from normal controls was identified, and increased miR-181b-5p expression levels were found to be associated with a poorer overall survival outcome. However, some of the highly expressed miRNAs were different from those found in previous studies. This inconsistency may be mainly due to differences in miRNA sources or to the difference between intracellular miRNAs and extracellular miRNAs. Other factors, such as study design, race, sample size or methodology may have also influenced the final results. These findings may have implications in the understanding of AML, establishing management strategies and estimating prognosis.

## Materials and Methods

### Sample Collection

Between 2008 and 2011, 140 newly diagnosed AML patients before treatment and 135 normal adult donors from the Department of Hematology at Third Affiliated Hospital of Soochow University were enrolled in this study with informed consent. The diagnosis of AML was established according to the criteria of the French-American-British (FAB) classification system based on standard morphological and immunophenotypic methods and all of the 140 AML patients were classified as M1 to M5 subtypes. Blood samples of all the patients were drawn within 24 hours after a diagnosis of AML was made. The coagulated blood samples were collected in tubes containing a separating gel and clot activator and centrifuged at 3,000 rpm for 15 min at room temperature. Then, the supernatant was centrifuged at 15,000 rpm for 30 min to precipitate cell debris and was stored at −80°C until use. Among the 140 primary AML patients, 18, 46, 17, 18 and 41 individuals were classified as M1, M2, M3, M4, and M5, respectively. The 135 control participants were recruited from a large pool of individuals who showed no evidence of disease and were seeking a routine health checkup at the Healthy Physical Examination Centre of Third Affiliated Hospital of Soochow University. All 140 AML samples and 135 normal controls were randomly assigned to a training set (45 AML samples versus 40 normal controls) or to a validation set (95 AML samples versus 95 normal controls). The clinical and demographic characteristics of the AML patients and normal controls are summarized in Table 2. Written informed consent was obtained from all patients or their representatives before the study, which was approved by the Research Ethics Board of the Third Affiliated Hospital of Soochow University.

**Table 2:** Demographic and clinical features of the AML and normal subjects.

		Training Set		Validation Set			Training Set		Validation Set	
Variable		Norm	AML	Norm	AML	Variable	Norm	AML	Norm	AML
**Age** **(mean±SD)**		47.18±15.24	51.42±16.31	51.45±17.54	47.90±15.23	**CD2**				
<47		18	28	56	48	Negative		28		81
≥47		22	17	39	47	Positive		17		14
**Gender**						**CD5**				
Male		20	24	45	58	Negative		41		90
Female		20	21	40	37	Positive		4		5
**Hemoglobin**						**CD7**				
Male						Negative		28		79
<120		0	24	0	53	Positive		17		16
≥120		20	0	45	5	**CD10**				
Female						Negative		45		91
<110		0	21	0	35	Positive		0		4
≥110		20	1	40	2	**CD13**				
**White blood cell**						Negative		1		6
<4.0		0	17	0	40	Positive		44		89
4.0–10.0		40	6	95	14	**CD14**				
>10.0		0	22	0	41	Negative		30		65
**Platelet**						Positive		15		30
<100		0	44	0	85	**CD19**				
100–300		38	1	95	8	Negative		35		78
>300		2	0	0	2	Positive		10		17
**FAB Classification**						**CD22**				
M1			5		14	Negative		39		87
M2			13		33	Positive		6		8
M3			8		9	**CD33**				
M4			8		10	Negative		0		5
M5			11		29	Positive		45		90
**LDH**						**CD34**				
≤245			17		39	Negative		14		30
>245			28		56	Positive		31		65

### Solexa Sequencing and Bioinformatics Analysis

The serum samples from 20 AML patients (M1–M5, four for each subtype) and 20 normal controls (Norm) with similar age and sex distributions were pooled for their respective groups. TRIzol reagent (Invitrogen) was used to extract total RNA from each pool of serum samples using a previously described protocol with minor modifications [15]. The total RNA was purified directly for Solexa sequencing analysis using an Illumina Genome Analyzer (Illumina, San Diego, CA, USA) according to the manufacturer’s instructions [51]. Briefly, a pair of Solexa adaptors was ligated to the 5′- and 3′-ends of the total RNA, and the small RNA molecules were amplified for 17 cycles by reverse transcription PCR (RT-PCR) using an RT-PCR kit (Invitrogen). The fragments that were approximately 30 bp, which were composed of small RNA plus adaptors, were isolated from an agarose gel. The purified RNA was directly used for cluster generation and sequencing analysis using an Illumina Genome Analyzer (Illumina, San Diego, CA, USA) according to the manufacturer’s instructions. After the bioinformatics analysis, the remaining miRNAs were searched against the Sanger miRBase (version19.0) [52]. Then, the differences in the quantities of the known miRNAs between the two groups were determined by comparing the log_2_ ratio of the AML and Norm group copies. This work was performed at the Beijing Genomics Institute (BGI).

### Quantification of miRNAs by qRT-PCR Analysis

For the qRT-PCR assay, the total RNA was extracted from 100 µL of serum by phenol/chloroform purification and centrifugation in isopropyl alcohol, as previously reported [53]. The TaqMan microRNA assay (Applied Biosystems, Foster City, CA) was performed using a TaqMan PCR kit with the Applied Biosystems 7500 Sequence Detection System according to the manufacturer’s instructions with a minor modification, as previously reported [15]. Briefly, the reverse transcription reaction was performed in 10 µL mixture containing 2 µL of RNA extracted from the serum, 1 µL of 10 mM dNTPs, 0.5 µL of AMV reverse transcriptase (TaKaRa, Dalian, China), 1µL of a stem-loop RT primer (Applied Biosystems), 2 µL of 5× reverse transcription buffer and 3.5 µL of DEPC-treated water. For synthesis of cDNA, the reaction mixture was incubated at 16°C for 15 min, 42°C for 60 min, 85°C for 5 min, and then held at 4°C. Real-time PCR was then performed with 1 cycle of 95°C for 5 min, followed by 40 cycles of 95°C for 15 sec and 60°C for 1 min. The real-time PCR reaction was performed in a final volume of 20 µL containing 1 µL of cDNA, 0.3 µL of Taq polymerase, 0.33 µL of TaqMan probe, 1.2 µL of 25 mM MgCl_2_, 0.4 µL of 10 mM dNTPs, 2 µL of 10× PCR buffer, and 14.77 µL of DEPC-treated water. All reactions, including controls containing no template RNA, were performed in triplicate. The expression data for the miRNA were acquired and analyzed using the ABI PRISM 7500 Sequence Detection System and 7500 Software v2.0.1 (Applied Biosystems, Foster City, CA, USA). The resulting Cq values were determined using fixed threshold settings. Due to the lack of a consensus housekeeping miRNA for qRT-PCR analysis of serum miRNA, we selected a normalization method utilizing the comparison of the miRNA concentration to the serum volume, as was described in our previous report.

### Statistical Analysis

The data are presented as the mean ± SEM for miRNAs or the mean ± SD for other variables. Student’s *t*-test or a two-sided chi-square test was used to compare differences in the serum miRNA concentrations between the two groups. Comparisons between more than two groups were performed using one-way ANOVA, and the differences between groups were subsequently determined by the Fisher LSD test when appropriate. A p-value <0.05 was considered statistically significant. We used Cluster 3.0 software (version 3.0.111.0, Stanford University, http://rana.stanford.edu/software) with the complete linkage method to perform hierarchical clustering. For each miRNA, we constructed the receiver operating characteristic (ROC) curve and calculated the area under the ROC curve (AUC) to evaluate its predictive power for AML.

## Supporting Information

Figure S1 **Cluster analysis of miRNA that are differentially expressed between AML patients and normal subjects.** For the training set (A), the validation set (B), and all samples (C), the miRNA expression levels in each group measured by qRT-PCR were normalized, mean-centered, clustered, and plotted as a heat map. The dendrogram generated by the cluster analysis show a clear separation of the AML from the normal subjects based on the 11 or 6 miRNA profiles.
(TIF)

Table S1 **Differentially expressed miRNAs in AML samples compared to normal subjects by Solexa sequencing.**
(XLS)

Table S2 **microRNA differentially expressed in AML subtypes from M1 to M5 compared to normal controls.**
(XLS)
