# Supplementary figures and images for: Identification of Circulating MicroRNAs as Potential Biomarkers for Detecting Acute Myeloid Leukemia
Source: PLoS One. 2013 Feb 20;8(2):e56718. doi: 10.1371/journal.pone.0056718 (PMC3577716; doi:10.1371/journal.pone.0056718)

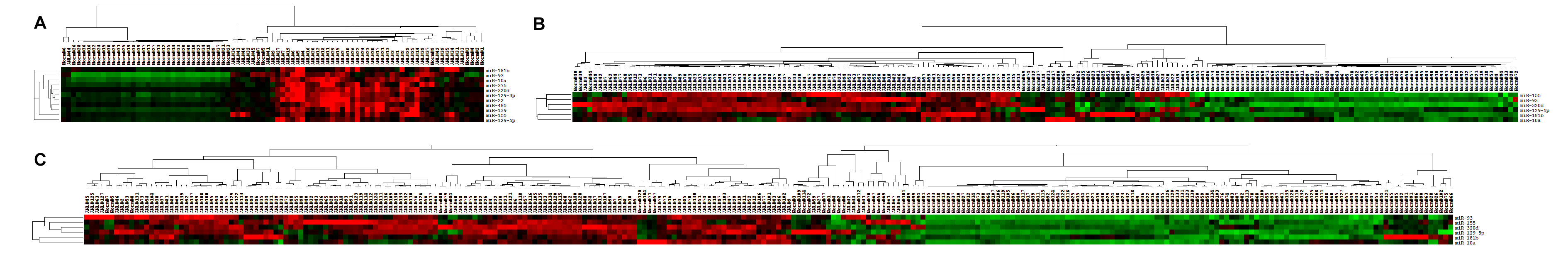

Supplement: Figure S1 — Cluster analysis of miRNA that are differentially expressed between AML patients and normal subjects. For the training set (A), the validation set (B), and all samples (C), the miRNA expression levels in each group measured by qRT-PCR were normalized, mean-centered, clustered, and plotted as a heat map. The dendrogram generated by the cluster analysis show a clear separation of the AML from the normal subjects based on the 11 or 6 miRNA profiles. (TIF) [file pone.0056718.s001.tif]
